# Phase Delay of the 40 Hz Auditory Steady-State Response Localizes to Left Auditory Cortex in Schizophrenia

**DOI:** 10.1177/15500594221130896

**Published:** 2022-10-10

**Authors:** Brian J. Roach, Yoji Hirano, Judith M. Ford, Kevin M. Spencer, Daniel H. Mathalon

**Affiliations:** 1 Mental Health Service, Veterans Affairs San Francisco Healthcare System, San Francisco, USA; 2 Northern California Institute for Research and Education (NCIRE), San Francisco, USA; 3 Neural Dynamics Laboratory, Research Service, Veterans Affairs Boston Healthcare System, Boston, USA; 4 Department of Psychiatry, 1811 Harvard Medical School, Boston, USA; 5 Department of Neuropsychiatry, Graduate School of Medical Sciences, Kyushu University, Fukuoka, Japan; 6 Department of Psychiatry and Behavioral Sciences and Weill Institute for Neurosciences, University of California at San Francisco, San Francisco, USA

**Keywords:** gamma, schizophrenia, EEG, oscillations, phase, ASSR (auditory steady-state response)

## Abstract

*Background.* The auditory steady state response (ASSR) is generated in bilateral auditory cortex and is the most used electroencephalographic (EEG) or magnetoencephalographic measure of gamma band abnormalities in schizophrenia. While the finding of reduced 40-Hz ASSR power and phase consistency in schizophrenia have been replicated many times, the 40-Hz ASSR phase locking angle (PLA), which assesses oscillation latency or phase delay, has rarely been examined. Furthermore, whether 40-Hz ASSR phase delay in schizophrenia is lateralized or common to left and right auditory cortical generators is unknown. *Methods*. Previously analyzed EEG data recorded from 24 schizophrenia patients and 24 healthy controls presented with 20-, 30-, and 40-Hz click trains to elicit ASSRs were re-analyzed to assess PLA in source space. Dipole moments in the right and left hemisphere were used to assess both frequency and hemisphere specificity of ASSR phase delay in schizophrenia. *Results.* Schizophrenia patients exhibited significantly reduced (ie, phase delayed) 40-Hz PLA in the left, but not the right, hemisphere, but their 20- and 30-Hz PLA values were normal. This left-lateralized 40-Hz phase delay was unrelated to symptoms or to previously reported left-lateralized PLF reductions in the schizophrenia patients. *Conclusions.* Consistent with sensor-based studies, the 40-Hz ASSR source-localized to left, but not right, auditory cortex was phase delayed in schizophrenia. Consistent with prior studies showing left temporal lobe volume deficits in schizophrenia, our findings suggest sluggish entrainment to 40-Hz auditory stimulation specific to left auditory cortex that are distinct from well-established deficits in gamma ASSR power and phase synchrony.

## Introduction

The auditory steady-state response (ASSR) is measured with electroencephalography (EEG) or magnetoencephalography (MEG) recordings by repeating a sound at a fixed repetition rate (eg, 40 times per second) and measuring the brain's response at that frequency (eg, 40-Hz). The ASSR is maximal when driven at a 40-Hz stimulation rate in humans,^1^ consistent with the status of gamma oscillations as a resonant frequency in the human brain. Gamma band (30-80Hz) oscillations are generated by recurrent inhibition from fast-spiking, parvalbumin-expressing GABAergic interneurons onto excitatory pyramidal neurons.^2–4^ N­-methyl-D-aspartate (NMDA) receptor hypofunction is a major pathophysiological mechanism in schizophrenia theorized to underlie cognitive and perceptual abnormalities of the illness.^5,6^ Because of the critical role of NMDA receptor glutamatergic signaling in these interneuron-pyramidal neuron microcircuits,^7^ deficits in 40-Hz ASSR power and phase-synchrony^8^ in schizophrenia are thought to arise, at least in part, from NMDA receptor hypofunction.

The vast majority of studies showing deficits in the 40-Hz ASSR in schizophrenia have used scalp-based sensor- (or electrode-) level EEG data rather than source-based MEG or EEG data (reviewed in^8^). However, source analyses of MEG data led to important, replicated discoveries about the 40-Hz ASSR: it has bilateral generators in primary auditory cortex^9–12^ and is greater in the right hemisphere than the left.^13,14^ The few source analytic studies of 40-Hz ASSR in schizophrenia published to date have demonstrated abnormalities in the location of bilateral cortical sources,^15^ reduction of the normal right greater than left hemisphere ASSR asymmetry,^16^ and reduced 40-Hz ASSR power or phase consistency specific to either the left,^17,18^ right,^16,19^ or both,^20,21^ hemispheres. ASSR abnormalities in schizophrenia could be further elucidated through source space analysis because of its ability to isolate left and right auditory cortical responses.

Whether an ASSR study uses sensor or source data, the same time-frequency (TF) measures of oscillation power and phase can be used to generate summary scores for statistical analysis. Evoked power, calculated from the cross-trial averaged ASSR time-domain waveform, and total power, calculated from single trial EEG epochs, are two measures of oscillation magnitude. Another common ASSR summary measure is the phase-locking factor (PLF), which measures the consistency, or lack of variability, in oscillation phase with respect to stimulus onset across single trials. While these three measures capture different aspects of EEG/MEG frequency domain signals over time,^22^ their 40-Hz ASSR values are highly correlated.^23,24^ An alternative time-frequency measure that captures the mean oscillation phase angle across trials is the phase-locking angle (PLA), which describes the degree to which an oscillation latency leads or lags the oscillation from a reference group.^25^ Phase delay in response to a stimulus could indicate abnormal GABAergic or glutamatergic signaling, abnormal subcortical-cortical communication, or other structural/functional brain abnormalities, but basic animal studies establishing these links are currently lacking. The 40-Hz ASSR PLA has excellent test-retest reliability that is comparable to the reliability of the 40-Hz ASSR PLF and power in both schizophrenia patients and healthy controls (^24^ but see Jiao et al ^26^). Previously, we showed that patients with schizophrenia have delayed 40-Hz ASSR relative to controls based on PLA calculated from scalp EEG.^23^ The relatively novel 40-Hz PLA measure has never been evaluated and compared between patients with schizophrenia and healthy controls in source space.

By measuring the phase lag of 40-Hz ASSR oscillations using PLA, we examined whether our previous finding of delayed 40-Hz ASSR in schizophrenia could be replicated and extended into EEG source space. Specifically, using 40-Hz ASSR EEG source waveforms generated in a prior study^17^ from patients with schizophrenia (SZ) and age-matched healthy controls (HC), we asked whether (1) SZ patients have delayed PLA in the gamma band response specific to 40-Hz auditory stimulation relative to HC; (2) delayed PLA in SZ is lateralized or evident bilaterally in primary auditory cortical dipole sources; (3) delayed PLA in SZ is specific to dipole moments in auditory cortex that are tangentially or radially oriented to the scalp. Delayed PLA was expected to be specific to tangential dipole sources given the previously observed phase delay in SZ at electrode Fz. Given that most prior ASSR EEG studies in SZ analyzed sensor level data without assessing PLA, the evidence that the 40-Hz ASSR PLA is delayed in schizophrenia in sensor space is quite limited. Accordingly, we also conducted supplementary analyses comparing HC and SZ on 40-Hz PLA assessed from scalp sensors. We expected that our prior finding of 40-Hz ASSR phase delay at electrode Fz in SZ^25^ would be replicated, and that any lateralized phase delay observed in source space would also be evident to some degree in the scalp sensors overlaying the corresponding hemisphere.

## Methods and Materials

### Participants

EEG data from a previously published study^17^ of 24 patients with schizophrenia and 24 HC were analyzed in the present study. The demographic and clinical data for these participants are summarized in Table 1^27^.

**Table 1.** Participant Demographic and Clinical Characteristics.

Variable	HC (N = 24)	SZ (N = 24)	Statistic	* P*
Age [years]	44.1 (7.3)	46.0 (9.1)	*t* [46] = −0.78	.439
Male / Female	20/4	20/4	*X²*(1) = 0	1.000
Handedness ^a^	0.79 (0.2)	0.81 (0.2)	*t* [46] = −0.38	.710
Self SES ^b^	2.25 (0.8)	3.42 (1.2)	*t* [39] = −3.72	.001^**^
Parental SES ^c^	2.46 (0.9)	2.83 (1.4)	*t* [41] = −1.11	.275
Education [years]	14.2 (1.7)	13.5 (2.0)	*t* [46] = 1.33	.188
MMSE ^d^	28.8 (1.4)	28.9 (1.4)	*t* [46] = −0.51	.615
WAIS-IV information subscale ^e^	10.7 (2.6)	10.1 (2.7)	*t* [46] = 0.87	.387
Duration of illness [years]		21.1 (9.7)		
Medication dosage [CPZ equiv, mg] ^f^		426.39 (444.03)		
Medication type [AP / TP / TP + AP / Non] ^g^		17 / 1 / 4 / 2		
SAPS - Positive Symptom Total ^h^		9.33 (4.90)		
SANS - Negative Symptom Total ^i^		9.38 (5.64)		

HC: healthy controls; SZ: schizophrenia patients.

^a^ Handedness was measured by Edinburgh Handedness Inventory. ^b,c^SES: Socioeconomic Status, Higher scores indicate lower SES. ^d^MMSE: Mini-Mental State Examination. ^e^WAIS-IV: Wechsler Adult Intelligence Scale-Fourth Edition. ^f^CPZ equiv: chlorpromazine equivalents. ^g^TP: typical antipsychotics, AP: atypical antipsychotics, Non: non-medicated patient. ^h^ SAPS: Scale for the Assessment of Positive Symptoms. ^i^ SANS: Scale for the Assessment of Negative Symptoms. Mean (SD) are given for each variable. Asterisks (*) indicate statistically significant results: ** *P* < .01.

All participants provided written, informed consent to participate in this [anonymized for peer review] IRB-approved study. Participants were excluded for (1) a history of electroconvulsive therapy, (2) history of major head trauma or neurological illness including epilepsy, (3) history of substance dependence or abuse within the past five years, (4) history of steroid use, and (5) Wechsler Adult Intelligence Scale-IV^28^ estimated premorbid intelligence quotient below 75. All participants were right-handed and screened for hearing loss. Patients met Diagnostic and Statistical Manual of Mental Disorders (DSM)-IV^29^ criteria for schizophrenia based on the Structured Interview for DSM-IV (SCID) Patient edition^30^ administered by a trained interviewer. Symptoms were rated using the Scale for the Assessment of Negative Symptoms (SANS;^31^) and the Scale for the Assessment of Positive Symptoms (SAPS;^32^). Potential HC participants were excluded for any personal history of an Axis-I disorder based on the SCID Non-Patient edition^30^ or for any family history of an Axis-I psychotic disorder in their first-degree relatives.

### ASSR Task

Participants were presented with three blocks of click trains (500ms duration, 800ms inter-train interval) binaurally through headphones at 70 dB sound pressure level. The click train rates were 20-, 30-, and 40-Hz, each presented within individual blocks of 150 trials, with the order of blocks counterbalanced across participants. All participants were instructed to view a fixation cross presented on a monitor and listen to the stimuli.

### EEG Recording, Processing, and Source Analysis

**Figure 1.**
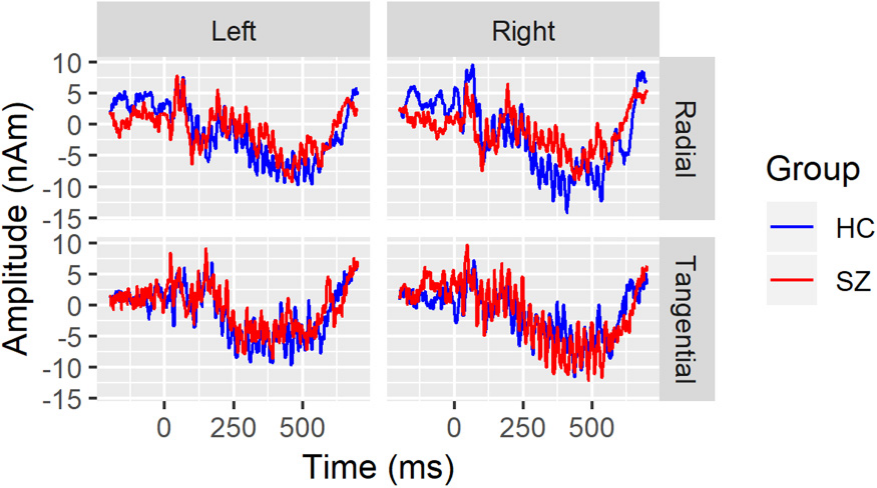
Grand average 40-Hz ASSR source waveforms are overlaid for healthy controls (HC; blue) and schizophrenia patients (SZ; red) separately for the left hemisphere (left column), right hemisphere (right column), radial (top row), and tangential dipoles. Time (in milliseconds) is plotted on the x-axis and amplitude (in nano-Ampere X meter) is plotted on the y-axis.

These procedures have been previously described in detail (see supplementary online content^17^), so are only briefly summarized here. EEG data were continuously digitized at 512Hz from 71 channels using a BioSemi ActiveTwo system (www.biosemi.com). A 0.1 Hz high-pass filter was applied before extracting 1300 ms epochs (−500 ms pre-stimulus to 800 ms post-stimulus onset) in BrainVision Analyzer 2.0.1 (Brain Products GmbH). Additional processing in MATLAB (Mathworks, Inc.) and Interactive Data Language (Exelis Visual Information Solutions) included artifact removal using independent components analysis,^33–36^ rejection of single trials containing amplitude artifacts, and re-referencing to the common average. Dipole source localization of the ASSR was done with Brain Electric Source Analysis (BESA) software v5.1.8 (BESA, GmbH). Similar to other dipole models of auditory cortex activity,^21,37,38^ a four-shell (scalp, skull, cerebrospinal fluid, and brain) spherical head model was fit to 13-100 Hz band-pass filtered HC grand average time domain waveforms and used to localize 2 dipoles in the superior temporal plane of each hemisphere. Dipoles were constrained to be symmetrically located but allowed to have free orientations. One pair was oriented tangentially to the lateral scalp (referred to as “tangential”) while the other pair had more radial orientations (referred to as “radial”). The final model accounted for 84.5% of the variance in the grand average 40-Hz ASSR waveforms between 30 and 530ms post click-train onset. Figure 1 shows the 40-Hz ASSR grand average source waveforms, separated by hemisphere and dipole orientation.

### Time-Frequency Analyses

TF analysis of EEG single trial data was done with a Morlet wavelet decomposition using freely distributed FieldTrip^39^ software in MATLAB. Specifically, a Morlet wavelet with a Gaussian shape defined by the ratio, f/σ_f_ = 6, was used, resulting in a 17.14 Hz spectral bandwidth at the 40-Hz frequency (ie, 31.43 to 48.57 Hz). Complex numbers from the wavelet decomposition are used to calculate the phase-locking factor (PLF,^40^) by normalizing the magnitude of each single trial value to the unit circle and averaging across trials, separately for each time point. The magnitude of that average complex number is equal to one minus the circular phase variance, and this measure is the PLF.^41^ The phase angle of that average is a measure of the latency or average location of the oscillation within a 360° cycle, and this measure is the PLA.^25^ The PLA, like other TF measures, is estimated from complex numbers and then converted to radians for subsequent processing using the circular statistics toolbox in matlab,^42^ and similar libraries are available in R.^43^

### Statistical Analysis

PLA values were extracted from each hemisphere and dipole between 30 and 530ms. Due to the periodic nature of the angle values, averaging over an interval equal to or greater than one complete sine wave cycle (eg, 25 ms for one 40-Hz cycle) will effectively cancel out individual values from the full, 360° cycle of phase angle measures, leading to an uninterpretable mean angle measure. Additional examples and illustrations of the differences between circular, phase angle measures and linear measures have been described previously (see Supplemental material in^25^). Therefore, the value of the phase angle at each time point is re-expressed as its difference from the expected angle at that time point. The expected angle in this study was defined as the HC circular mean at a specific time point in the right tangential dipole, where the evoked response was strongest.^17^ For sensor level comparisons, the expected angle was defined using the HC circular mean at electrode Fz. This difference defines the PLA, and it is expressed in units of radians. The average PLA at 40-Hz between 30 and 530ms was used for subsequent analysis of the 40-Hz ASSR. Similar PLA calculations were applied to the 20-Hz and 30-Hz ASSR elicited during the 20- and 30-Hz click train blocks, respectively. Use of a single dipole orientation for calculating the PLA allowed for differences in hemisphere, dipole orientation, and their interaction to be assessed in statistical analysis. Due to the periodic, non-linear nature of PLA data, PLA values were z-scored by calculating the phase difference from the right tangential HC circular mean and dividing by the right tangential HC circular standard deviation to facilitate linear modeling. Without performing a circular-to-linear z-score transformation, analyses of phase angle data would be restricted to simple circular statistics methods (eg, Watson-Williams test, circular-linear correlations, etc) that (i) limit interpretation of the results due to lack of signed correlations or test statistics, (ii) prohibit the use of repeated measures or other, modern statistical methods, and (iii) do not generate effect size estimates (ie, Cohen's d) that allow for comparisons with PLF or power ASSR measures from the same data set or from other published studies. Note, the sum of the differences between the circular mean phase angle and each observation that contributes to it will not always equal zero. Thus, the mean of z-scored PLA values is not always zero, and z-scoring converts this circular measure to a linear measure, in units of standard deviation from the HC mean.

To assess possible differential sensitivity to the pathophysiology of schizophrenia, 20-Hz, 30-Hz, and 40-Hz PLA z-scores were tested in a mixed model with Group as the between-subjects fixed factor and Hemisphere (Right, Left), Orientation (Tangential, Radial), and Frequency (20-Hz, 30-Hz, 40-Hz) as the within-subjects fixed factors. Subject nested within Group was a random factor and the main effects, interaction, and follow-up contrasts were estimated with an unstructured covariance matrix using proc glimmix in SAS v9.4. Any significant Group x within-subject factor interactions in the mixed model were followed up with Tukey-Kramer adjusted tests of the SZ versus HC contrast.

To assess possible group and topographical differences in 40-Hz ASSR PLA z-scores at the sensor level, an additional mixed model was run including factors for Hemisphere (Left, Midline, Right) and Region (Frontal, Frontocentral, Frontotemporal, Central, and Parietal). Electrodes F1, F3, FC1, FC3, C1, C3, FT9, P9, F2, F4, FC2, FC4, C2, C4, FT10, and P10 were included.

To examine relationships between clinical symptoms and PLA measures in the SZ group, we conducted Pearson correlations between SAPS and SANS global total symptom scores and 40-Hz PLA z-scores from source-space data.

## Results

Following artifact rejection, HC retained 140 single trials (SD = 13) for the 20-Hz block and 139 single trials (30-Hz SD = 14, 40-Hz SD = 12) for the other blocks, on average. SZ retained 135 trials for 20-Hz and 30-Hz blocks (20-Hz SD = 13, 30-Hz SD = 14) and 137 single trials for the 40-Hz block (SD = 13). Visual inspection of box plots of the z-scored data did not reveal any outliers.

### Mixed Models

**Figure 2.**
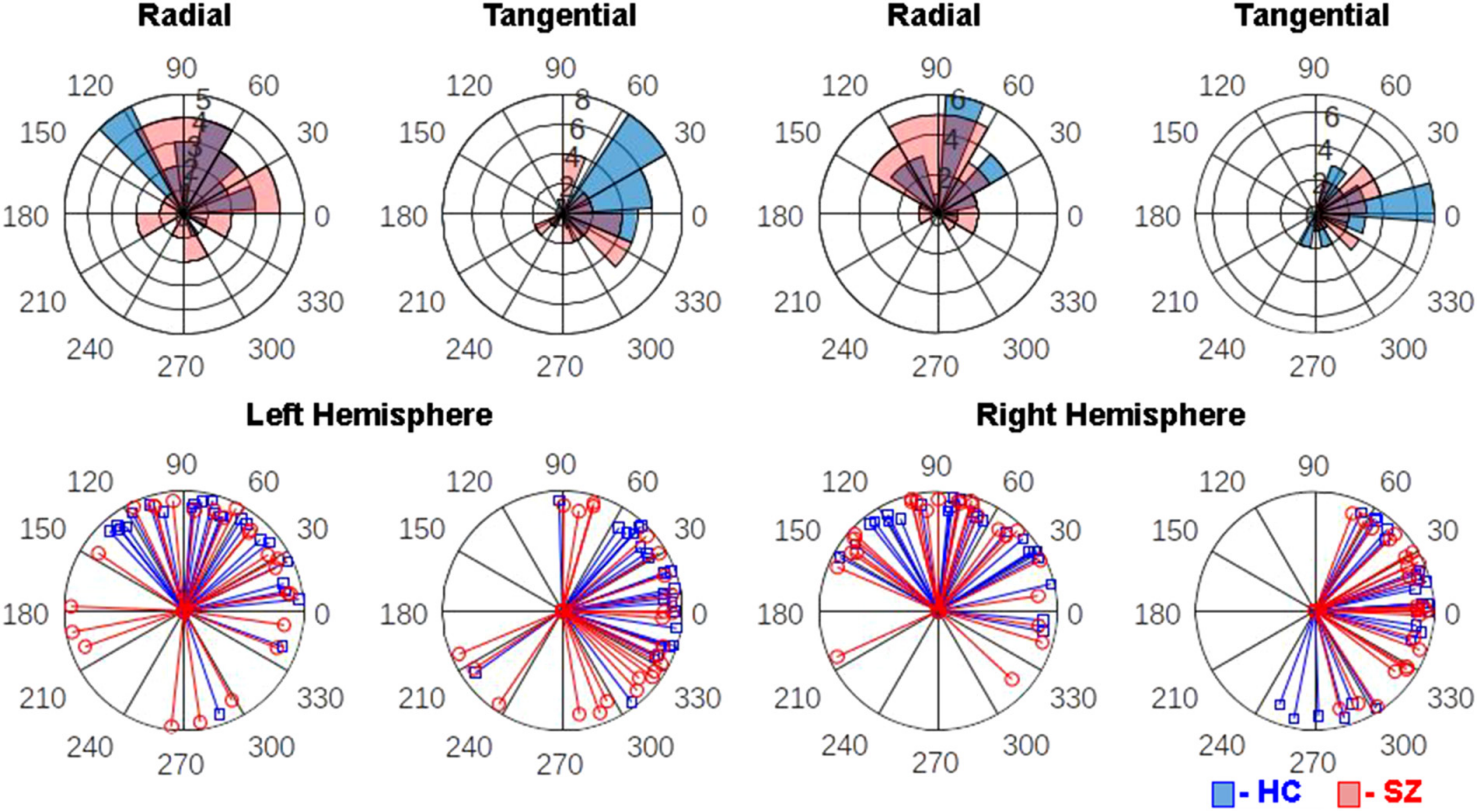
Polar histogram overlays depict the distribution of 40-Hz ASSR phase-locking angle (PLA) measures in healthy controls (HC, blue) and schizophrenia patients (SZ, red) in the left hemisphere radial dipole (top far-left), left hemisphere tangential dipole (top center-left), right hemisphere radial dipole (top center-right), and right hemisphere tangential dipole (top far-right). On these histograms, each wedge represents a phase angle bin and its length indicates the number of subjects who fall into that bin. Single subject PLA vectors are plotted for the same hemisphere and dipole combinations in the bottom row, with each blue line representing one HC and each red line representing one SZ.

**Figure 3.**
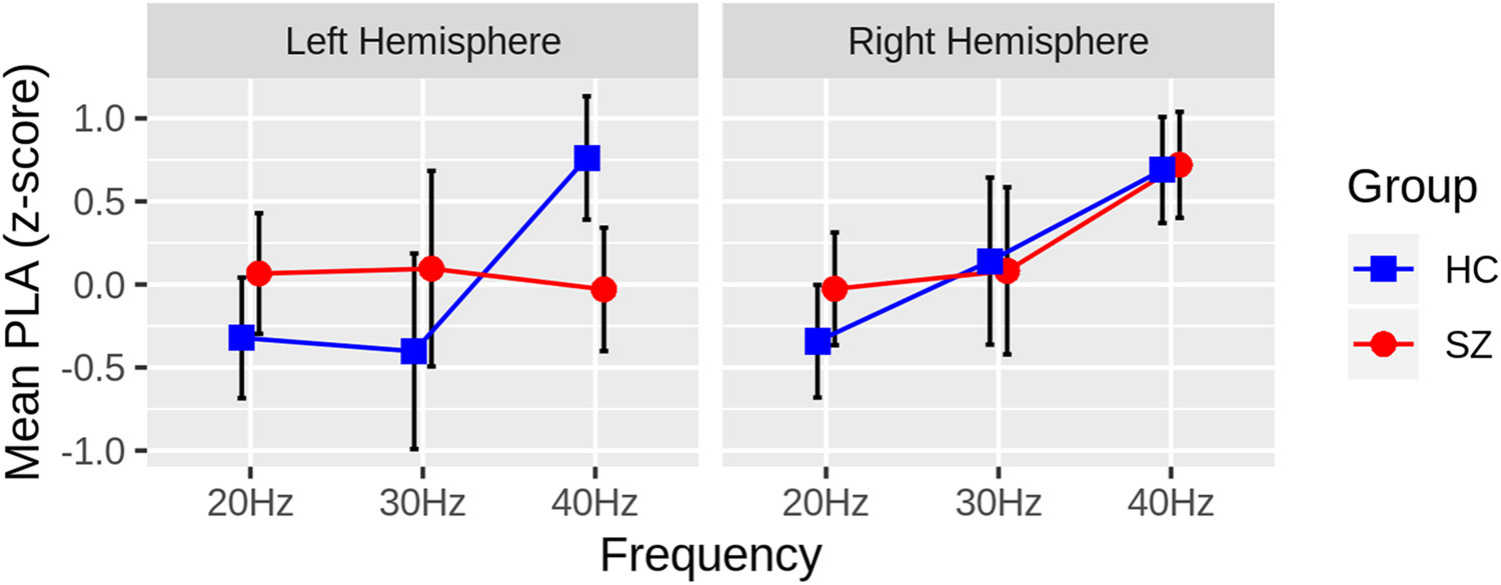
Group differences in the ASSR phase-locking angle (PLA) z-scores are plotted for each hemisphere and steady-state frequency (Hz), collapsing across radial and tangential dipoles. The Healthy Controls (HC: blue squares) have greater PLA z-scores than the Schizophrenia Patients (SZ: red circles) in the left hemisphere, 40Hz ASSR only, indicating that SZ lag behind HC in the left hemisphere. Black bars depict 95% confidence intervals for each measure.

In the mixed model of PLA z-scores, there was a significant Group X Hemisphere X Frequency interaction (F(2,46) = 3.88, *P* = .0276). Follow-up contrasts revealed that SZ significantly lagged behind HC 40-Hz ASSR in the left (t(46) = 3.03, *P* = .004, adj-*P* = .0431) but not the right hemisphere (t(46) = −0.14, *P* = .89). There were no differences between groups in 20-Hz or 30-Hz ASSR in either hemisphere (ps > .135). This pattern of effects can be seen before z-scoring (Figure 2) and following z-scoring (Figure 3) of the PLA values. There were no other Group interaction effects, and the main effect of Group was not significant (F(1,46) = 0.33, *P* = .57). There were main effects of Frequency (F(2,46) = 10.17, *P* = .0002) and Orientation (F(1,46) = 13.31, *P* = .0007) and a Frequency X Orientation interaction (F(2,46) = 13.32, *P *< .0001). These main effects were driven by a linear increase (ie, 20-Hz<30-Hz<40-Hz) in PLA as ASSR frequency increased and the radial PLA leading the tangential PLA dipoles. The interaction effect was mainly a function of radial leading tangential dipoles at 40-Hz (t(46) = 5.75, *P* < .0001) but not at the other ASSR frequencies (ps > .09).

The sensor data mixed model showed no significant main effects or interactions, although there was a trend level main effect of Hemisphere due to delay in the right relative to left hemisphere electrodes (t(46) = 1.68, *P* = .094). The lack of a group difference was unexpected, and an additional exploratory t-test comparing groups at electrode Fz was conducted, revealing no difference in PLA z-scores (t(46) = −0.028, *P* = .977).

### Post-hoc Assessment of Phase-Locking Angle Relationship with Phase-Locking Factor

**Figure 4.**
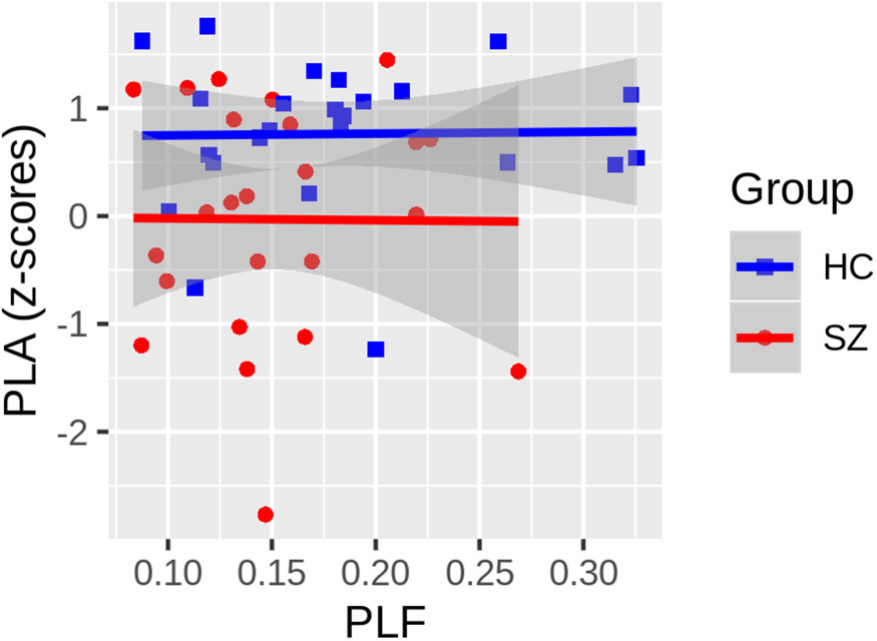
The relationship between the ASSR phase-locking angle (PLA) z-scores and phase-locking factor, each averaged across radial and tangential dipoles, are plotted. Neither the Healthy Controls (HC: blue squares) nor the Schizophrenia Patients (SZ: red circles) show any relationship between the PLA and PLF measures, and there was no significant difference between the slope of the regression lines fit within each group (t(44) = -0.069, *P* = .946). While not shown, the common slope, collapsing across groups, was also non-significant (t(45) = 0.025, *P* = .98). Gray shading around regression lines depict 95% confidence intervals for each group.

Given the PLA deficits in the left hemisphere and previously reported PLF abnormalities in the left hemisphere of this SZ patient sample, a post-hoc evaluation of the relationship between these measures was conducted. To determine if the left-lateralized phase delay could be accounted for by the previously reported, left-lateralized PLF deficit in SZ, a General Linear Model (GLM) was implemented with Group and PLF as regressors. Before testing the common slope across groups, PLF × Group interaction terms were included in a higher-order GLM to test for significant slope differences between groups. If the interaction term did not significantly improve model fit, HC versus SZ slope differences were assumed not to exist, and the simplified GLM was used to predict PLA. This GLM also provides analysis of covariance-style tests of group differences in PLA, controlling for PLF. There was no PLF x Group interaction (t(44) = -0.069, *P* = .946), indicating that the relationship between the left hemisphere PLA z-scores and PLF did not differ between groups (Figure 4). There was no correlation between PLA and PLF in the reduced model (t(45) = 0.025, *P* = .98), and the delayed PLA in SZ remained statistically significant (t(45) = -2.891, *P* = .0059), controlling for PLF.

### Clinical Correlations

There were no symptom correlations with 40-Hz PLA z-scores for either of the left or right hemisphere sources (ps > 0.185).

## Discussion

This study compared the 20-Hz, 30-Hz, and 40-Hz ASSR PLA in radial and tangential dipoles located in bilateral auditory cortex in SZ and HC and replicated the finding that SZ patients have delayed PLA in the gamma band ASSR specific to 40-Hz auditory stimulation relative to HC. This delayed 40-Hz PLA in SZ was present only in the left primary auditory cortex source activity. There was no evidence that delayed PLA was specific to tangential dipole sources, which was unexpected but consistent with the lack of significant phase delay in SZ at electrode Fz. There were no clinical symptom correlations with 40-Hz ASSR phase delay in SZ. At the sensor level, this study failed to replicate previous findings of strong phase delay in SZ relative to HC.

While this is only the second study to report phase delay in SZ based on the PLA measure, both the current and prior^23^ study are consistent with the first report of 40-Hz ASSR abnormalities in SZ, where Kwon et al reported group differences on an alternative measure of phase delay.^44^ Like the prior PLA study, the group difference was a larger effect for PLA (Cohen's d = 0.895) than PLF (d = 0.54). Neither study observed a significant relationship between PLA and positive or negative symptom scores, possibly reflecting the many methodological challenges associated with demonstrating symptom correlations with biomarker measures in schizophrenia.^45^ This includes deficient power to detect medium or small effects when sample sizes are small, as in the present study. Additional, larger sample studies are needed to identify any clinical and potential cognitive correlates of PLA abnormalities in SZ.

The application of BESA allowed 64 channel scalp EEG data to be analyzed in source space. Estimation of 40-Hz ASSR activity in left and right hemisphere auditory cortical sources allowed us to identify a left-lateralized 40-Hz ASSR phase delay in SZ that would not have been evident if analyses had been confined to scalp sensor data. Previous studies have established that bilateral generators in primary auditory cortex are the main source of the ASSR,^9–12^ and there is normally greater 40-Hz ASSR in the right compared to the left hemisphere.^14^ Reduced gray matter volume in the superior temporal gyrus (STG) has been demonstrated in SZ,^46,47^ and a meta-analysis suggests this decline is progressive,^48^ consistent with evidence that cortical gray matter decline in STG predates psychosis onset.^49–51^ Left temporal lobe gray matter deficits, in particular, have long been considered a potential critical component of schizophrenia pathophysiology.^52–54^ Attempts to associate STG volume or thickness with 40-Hz ASSR produced inconsistent results, showing left hemisphere STG – 40-Hz ASSR associations limited to either SZ^55^ or HC,^18^ but not both groups. In contrast to these studies, Kim et al showed right hemisphere STG volume was correlated with 40-Hz ASSR evoked power in HC only.^56^ Given such mixed results, it will be important to replicate the left hemisphere 40-Hz ASSR phase delay in a larger SZ sample study and consider both structural and functional (eg, thalamus-STG connectivity) anatomical correlates of phase delay.

The 4-dipole solution used in the current study provides a simple, spatial filter to reduce scalp EEG data dimensionality, but it is likely an incomplete model of ASSR generation in the brain, as subcortical generators may also be involved (eg, Wang, Li et al, 2020). It is challenging to measure and successfully reconstruct source activity from sub-cortical structures using scalp EEG data, but fMRI or other imaging modalities and non-human animal studies may provide additional insights into the phase delay in other nodes of the auditory network. Nonetheless, simple sensor-based analysis alone failed to demonstrate any phase delay in SZ in this study, and a comparison of Fz PLA z-scores did not replicate prior studies.^23^ This suggests source reconstructions are more sensitive to phase delay in SZ than simple sensor level data. One possible explanation is that the mixing of sources at the scalp level obscures phase delay effects that are present at the source level.

This study also replicates the frequency specificity of the phase delay abnormality in SZ, which was not present in the 20-Hz or 30-Hz ASSR. That the phase delay is restricted to the 40-Hz ASSR suggests that it could be an index of NMDA receptor and/or GABAergic neurotransmission abnormalities, given the known role for each in the generation of gamma oscillations.^2,3^ While EEG does not provide for a direct assessment of the cellular microcircuits that generate gamma oscillations, homologous pharmacological challenge studies in humans and invasive animal studies have some potential to bridge from EEG to underlying microcircuits, as the 40-Hz ASSR is easy to measure and can be recorded from different species.

The PLA is a measure of the mean phase angle relative to a reference or control group, and it can be considered an index of oscillation latency. The PLF measure is also derived from oscillation phase angles, but it captures the phase consistency, or lack of phase variability, across single trials. While it is tempting to speculate that the phase delay observed in SZ is a result of the increased phase variability (ie, decreased PLF), the left-lateralized phase delay in SZ was not accounted for by PLF, consistent with sensor-based analysis of the PLA abnormality in SZ persisting after controlling for PLF.^23^ Furthermore, there was no group-specific or common relationship between PLA and PLF measures, which is consistent with previous studies^23,24^ and provides more evidence that these two measures of oscillation phase, as well as their abnormalities in schizophrenia, are uncorrelated.

### Limitations

The patients in this study were taking antipsychotic medication, and like the majority of prior ASSR studies in schizophrenia and the prior report of PLA delay, confounding influences of medication cannot be ruled out. Studies of patients earlier in the illness course or unmedicated individuals at clinical high-risk for developing psychosis may provide additional information about the role of antipsychotic medications in 40-Hz ASSR phase delay. While the failure to detect clinical symptom correlations was not unexpected given the sample size, additional, well-powered studies are needed to provide more insight into the functional consequences of delayed latency of 40-Hz responses in schizophrenia. Additionally, a mechanistic understanding of gamma band phase delay in schizophrenia will require future basic neuroscience studies in animal models testing whether or not phase delay is a consequence of NMDA receptor hypofunction, abnormal GABAergic signaling, or some other underlying mechanism. Failure to replicate previously observed phase delay in SZ at electrode Fz suggests this simple analytic approach is inadequate and different sources may cancel or mask phase delays at particular scalp sites.

Patients in this study and most others show reduced PLF relative to controls. There is no generally accepted cut-off applied to the PLF to indicate a subject failed to produce an ASSR and should be excluded from analyses (eg, exclude subjects with PLF < 0.1). If such a threshold existed, it could be used to exclude subjects prior to analyzing PLA to ensure that the mean phase angles are not analyzed for subjects who failed to respond to the stimulus. If the goal of this or another study of patients and controls were only focused on comparing the PLA between groups in one condition (eg, 40-Hz) at a single time point and individual electrode or source, the Watson-Williams test compares phase angles (in radians) between two groups.^57^ It would be possible to test more time points and combine with a cluster-based permutation testing strategy,^58^ avoiding the need to convert phase angles from radians to z-scores.

### Conclusions

The 40-Hz PLA was significantly delayed in the left auditory cortex of patients with schizophrenia relative to healthy controls, irrespective of dipole orientation. While power measures the ASSR magnitude and PLF measures the consistency of phase across trials, PLA quantifies the mean phase angle and provides a continuous measure of oscillation latency that appears to be more sensitive to the pathophysiology of schizophrenia than power and PLF measures, based on our current findings and our prior report.^25^ As such, it could provide valuable, complementary insight into abnormalities in interneuron – pyramidal neuron network function in psychosis.
